# Supplementary material for: Parent Acceptance toward Inactivated COVID-19 Vaccination in Children with Acute Lymphoblastic Leukemia: The Power of Oncologist and Alliance
Source: Vaccines (Basel). 2022 Nov 25;10(12):2016. doi: 10.3390/vaccines10122016 (PMC9785446; doi:10.3390/vaccines10122016)
Supplement: Supplementary file 1 [file vaccines-10-02016-s001.zip › vaccines-2021241-supplementary.pdf]

Supplementary Materials

Additional Backgrounds

Severe acute respiratory syndrome coronavirus-2

Severe Acute respiratory syndrome Coronavirus 2 (SARS-CoV-2) infection and the resulting disease, COVID-19, first emerged in 2019, and WHO declared a pandemic in March 2020[1]. SARS-CoV-2 uses spikes (S) glycoproteins on the viral envelope to attach itself to respiratory cell surfaces that express host cell transmembrane serine protease 2 (TMPRSS2) mediated angiotensin-converting enzyme 2 (ACE2) receptors and S proteins [2, 3]. In addition to the respiratory tract, ACE2 is also expressed on the cells of different tissues, such as alveolar cells of the lung, muscle cells of the heart, and vascular endothelium. After attachment, it can enter cells and replicate in cells to cause disease [2, 3].

Individuals infected with SARS-CoV-2 exhibit a wide range of heterogeneous clinical manifestations, ranging from asymptomatic cases to severe disease that can lead to death [4]. Those at highest risk of severe illness and death include the elderly and those with pre-existing conditions such as cancer [5-7]. The physiology and pathology of COVID-19 involves complex host-viral interactions of different immune cells and inflammatory molecules. Unbalanced immune responses such as low responsiveness (uncontrolled viral replication) and high responsiveness (disproportionate inflammation) can lead to severe COVID-19[4]. Currently, there is a lack of effective treatment for immunocompromised patients, and the treatment is usually complementary therapy [8]. Therefore, the vaccine as a preventive measure can effectively reduce the risk of death in these patients.

COVID-19 vaccines

Similar to other vaccines, the novel coronavirus vaccine infuses the forged novel coronavirus antigen into the human body to make the immune system recognize the antigen and conduct immune attack, forming immune memory [9]. Specifically, if a person is infected, the vaccine will trigger an immune response that can block or kill the virus. Any subsequent COVID-19 antigens will be effectively recognized and attacked by memory immune cells to prevent damage from COVID-19. Researchers around the world have been working to develop a vaccine for COVID-19 since the outbreak began, with more than 198 vaccines currently in preclinical or clinical development. Frantic efforts in vaccine development have led to several vaccine candidates from multiple platforms that have entered the clinical evaluation stage, including inactivated vaccines, live virus vaccines, recombinant protein vaccines, vector vaccines, and DNA or RNA vaccines [11,12].

Currently approved vaccine types in China include 2 doses of inactivated vaccine (Sinovac and Sinopsin) and 1 dose of adenovirus vaccine (Ad5-nCoV). In phase I/II trials, both vaccines showed good immunogenicity and moderate adverse events in healthy people. Because cancer patients are usually immunocompromised, vaccines that carry live viruses are usually prohibited. Therefore, the COVID-19 vaccine recommended by oncologists in this study usually refers to inactivated vaccines.

Acute lymphoblastic leukemia

Acute lymphoblastic leukemia (ALL) is a malignant disease resulting from abnormal proliferation of B-line or T-line cells from bone marrow lymphocytes. ALL has been documented as the most common childhood malignancy, accounting for 25% of all childhood cancers [14]. Immunosuppression in patients with leukemia is either due to a disease state involving clonal

amplification of undifferentiated and functionally abnormal lymphoid progenitors. They invade the bone marrow, peripheral blood, and extramedullary sites [15] or are immunocompromised due to chemotherapy-induced immunosuppression. Immunocompromised patients are at high risk for viral reactivation or new viral infections [16].

Cell morphology, immunology, cytogenetics and molecular biology can be used to diagnose ALL. The treatment is usually combined with bone marrow transplantation and chemotherapy, and early treatment [17] can obtain a long-term survival prognosis. In the first wave of treatment, patients with hematological malignancies have a poor prognosis, with a mortality rate of 20-40%. The single or simultaneous activation of latent viruses can have serious consequences [19], so the prevention of viral infection is very important. However, severe immunosuppression is a key issue during or after treatment, for which many parents are hesitant to vaccinate their children against COVID-19.

References

1. WHO, Weekly epidemiological covid-19-6 july 2021 edition 47. https://www.who.int/publications/m/item/weekly-epidemiological-update-on-covid-19%2D%2D-6-july-2021. (Published Date 6 July 2021, Accessed Date 11 July 2021).
2. Walls AC, Park Y-J, Tortorici MA, et al. Structure, function, and antigenicity of the SARS-CoV-2 spike glycoprotein. Cell. 2020;181:281–292.e6.
3. Hoffmann M, Kleine-Weber H, Schroeder S, et al. SARS-CoV-2 cell entry depends on ACE2 and TMPRSS2 and is blocked by a clinically proven protease inhibitor. Cell. 2020;181:271–280.e8.
4. Docherty AB, Harrison EM, Green CA, et al. Features of 20 133 UK patients in hospital with covid-19 using the ISARIC WHO Clinical Characterisation Protocol: prospective observational cohort study. BMJ. 2020 May 22;369:m1985.
5. Williamson EJ, McDonald HI, Bhaskaran K, et al. Risks of covid-19 hospital admission and death for people with learning disability: population based cohort study using the OpenSAFELY platform. BMJ. 2021 Jul 14;374:n1592.
6. Williamson EJ, Walker AJ, Bhaskaran K, et al. Factors associated with COVID-19-related death using OpenSAFELY. Nature. 2020 Aug;584(7821):430-436.
7. Tizazu, A.M., Mengist, H.M., Demeke, G. Aging, inflammaging and immunosenescence as risk factors of severe COVID-19. Immun Ageing 19, 53 (2022).
8. Cook LB, O'Dell G, Vourvou E, et al. Third primary SARS-CoV-2 mRNA vaccines enhance antibody responses in most patients with haematological malignancies. Nat Commun. 2022 Nov 14;13(1):6922.
9. Callaway E. The race for coronavirus vaccines: a graphical guide. Nature. 2020 Apr;580(7805):576-577.
10. WHO . World Health Organization; Geneva: Oct 19, 2020. Draft landscape of COVID-19 candidate vaccines. https://www.who.int/publications/m/item/draft-landscape-of-covid-19-candidate-vaccines
11. Krammer F. SARS-CoV-2 vaccines in development. Nature. 2020;586:516–527.
12. Callaway E. The race for coronavirus vaccines: a graphical guide. Nature. 2020;580:576–577.
13. Zhang Y, Zeng G, Pan H, et al. Safety, tolerability, and immunogenicity of an inactivated SARS-CoV-2 vaccine in healthy adults aged 18-59 years: a randomised, double-blind, placebo-controlled, phase 1/2 clinical trial. Lancet Infect Dis. 2021 Feb;21(2):181-192.

14. Pui CH. Childhood leukemias. N Engl J Med. 1995 Jun 15;332(24):1618-30.
15. Roberts KG, Mullighan CG. The Biology of B-Progenitor Acute Lymphoblastic Leukemia. Cold Spring Harb Perspect Med. 2020 Jul 1;10(7):a034835.
16. Sehrawat S, Kumar D, Rouse BT. Herpesviruses: Harmonious Pathogens but Relevant Cofactors in Other Diseases? Front Cell Infect Microbiol. 2018 May 25;8:177.
17. Rouger-Gaudichon J, Bertrand Y, Boissel N, et al. COVID19 and acute lymphoblastic leukemias of children and adolescents: Updated recommendations (Version 2) of the Leukemia Committee of the French Society for the fight against Cancers and leukemias in children and adolescents (SFCE). Bull Cancer. 2021 May;108(5):490-500.
18. Cook, L.B., O’ Dell, G., Vourvou, E. et al. Third primary SARS-CoV-2 mRNA vaccines enhance antibody responses in most patients with haematological malignancies. Nat Commun 13, 6922 (2022).
19. Jaskula E, Dlubek D, Sedzimirska M, et al. Reactivations of cytomegalovirus, human herpes virus 6, and Epstein-Barr virus differ with respect to risk factors and clinical outcome after hematopoietic stem cell transplantation. Transplant Proc. 2010 Oct;42(8):3273-6.

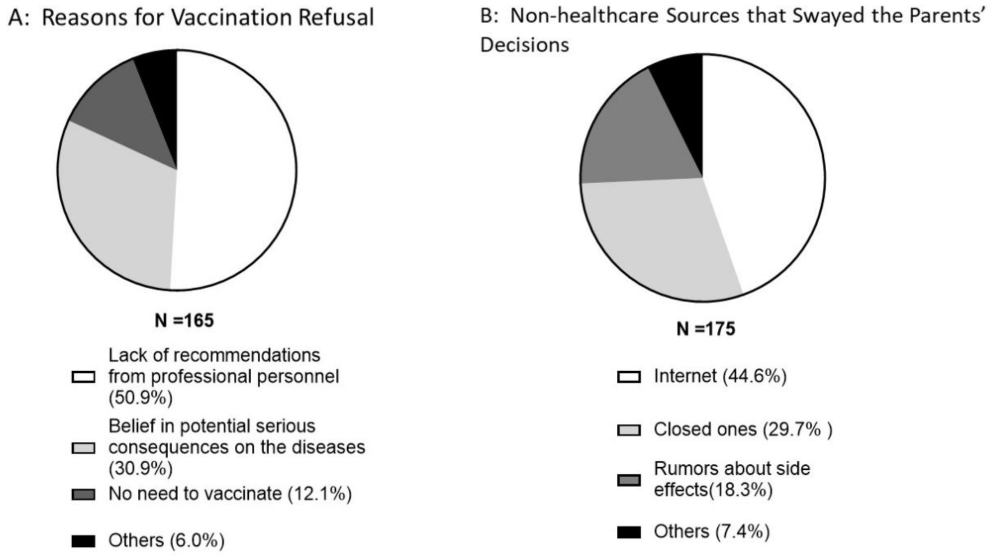

Supplementary Figure S1. Attitudes toward COVID-19 vaccination on CALLS during the interview with oncologists. 1A, reasons for vaccination refusal; 1B, non-healthcare sources that would sway the decision to vaccinate.

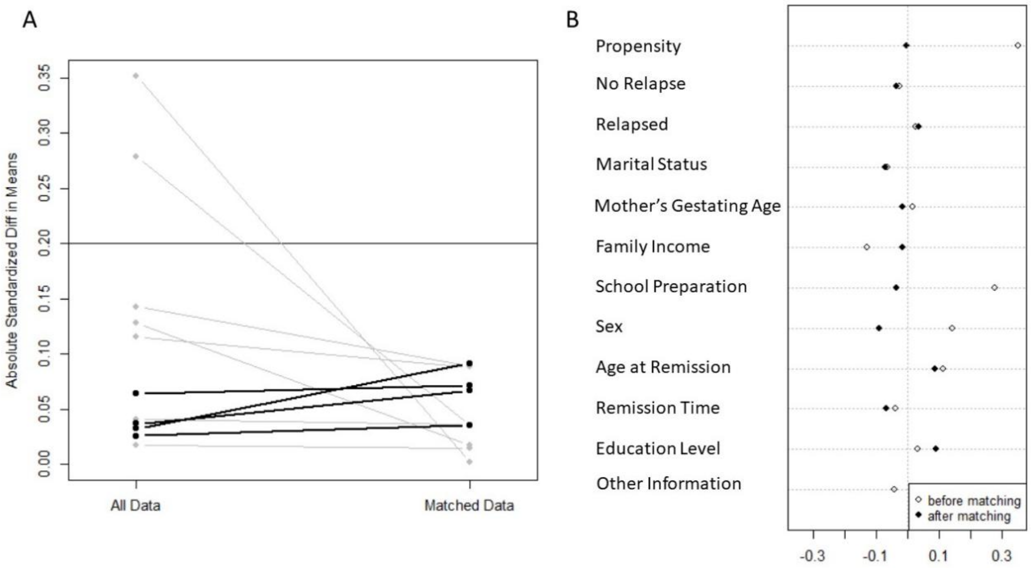

Supplementary Figure S2. Propensity score matching efficacy evaluation based on oncologist recommendation. 2A, absolute standardized differences (ASD) in unmatched and matched samples; 2B, dot plot of ASD of each variable that entered the matching. Mother's Gestating Age: Calculated as mother's age minus the age of the CALLS.

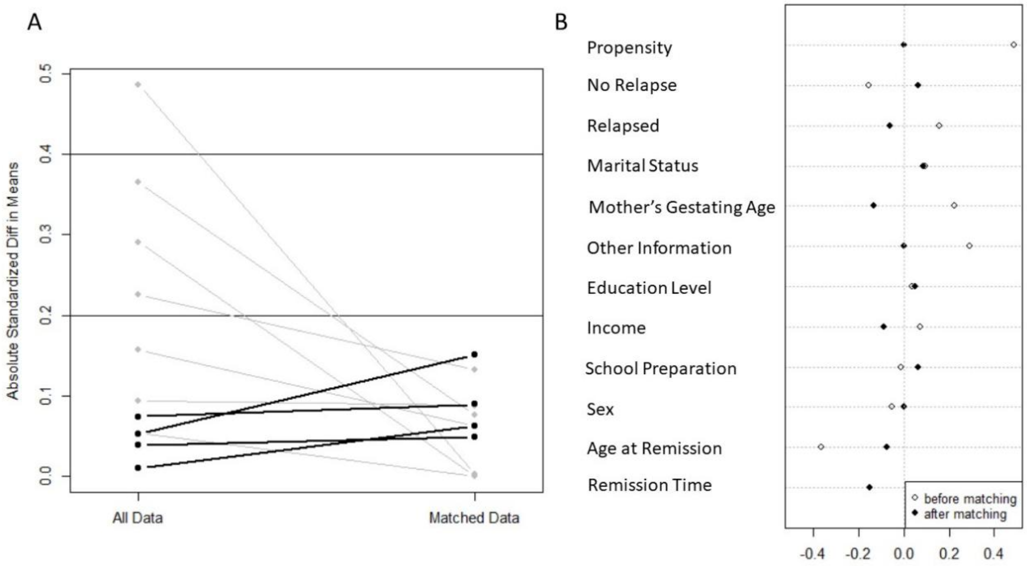

Supplementary Figure S3. Propensity score matching efficacy evaluation based on the parent-oncologist alliance. 3A, absolute standardized differences (ASD) in unmatched and matched samples; 3B, dot plot of ASD of each variable that entered the matching. Mother's Gestating Age: Calculated as mother's age minus the age of the CALLS.

Supplementary Table S1. Detailed Balance Test of Propensity Score Matching of Oncologist Recommendation vs. Control.

Covariates	Means Treated		Means Control		Standardized Mean Difference	
	Before	After	Before	After	Before	After
Propensity	0.3	0.29	0.27	0.29	0.35	0.00
Never relapsed	0.6	0.6	0.61	0.61	-0.03	-0.04
Ever relapsed	0.4	0.4	0.39	0.39	0.03	0.04
Marital Status	0.84	0.83	0.86	0.86	-0.06	-0.07
Mother's Gestating Age[a]	3.05	3.04	3.03	3.05	0.02	-0.01
Annual family income	1.74	1.74	1.87	1.75	-0.13	-0.02
School preparation	0.55	0.54	0.41	0.55	0.28	-0.04
Sex	0.59	0.58	0.52	0.62	0.14	-0.09
Age at Remission	6.28	6.25	5.88	5.95	0.12	0.09
Remission time	1.69	1.73	1.74	1.81	-0.04	-0.07
Highest education level of the family	2.42	2.42	2.40	2.36	0.03	0.09
Influenced by non-healthcare information	0.40	0.40	0.42	0.39	-0.04	0.04

[a]: Calculated as mother's age minus the age of the CALLS.

Supplementary Table S2. Detailed Balance Test of Propensity Score Matching of Low Patient-Oncologist Alliance Score vs. non-Alliance in the Recommendation Group.

Covariates	Means Treated		Means Control		Standardized Mean Difference	
	Before	After	Before	After	Before	After
Propensity	0.56	0.49	0.49	0.49	0.49	0.00
Never relapsed	0.56	0.63	0.64	0.59	-0.16	0.06
Ever relapsed	0.44	0.38	0.36	0.41	0.16	-0.06
Marital status	0.85	0.78	0.82	0.75	0.09	0.09
Mother's Gestating Age [a]	3.18	3	2.91	3.16	0.23	-0.13
Influenced by Non-healthcare Information	0.47	0.34	0.32	0.34	0.29	0.00
Highest education level of the family	2.44	2.41	2.41	2.38	0.04	0.05
Family income	1.77	1.66	1.7	1.75	0.07	-0.09
School preparation	0.55	0.53	0.55	0.5	-0.01	0.06
Sex	0.58	0.63	0.61	0.63	-0.05	0.00
Age at Remission	5.71	7.03	6.91	7.28	-0.36	-0.08
Remission time	1.73	1.59	1.66	1.78	0.05	-0.15

[a] Calculated as mother's age minus the age of the CALLS.
